# Functional group analyses of herpetofauna in South Korea using a large dataset

**DOI:** 10.1038/s41597-022-01924-z

**Published:** 2023-01-05

**Authors:** Jong Yoon Jeon, Dong Kun Lee, Jae Hyun Kim

**Affiliations:** 1 Research Institute of Agriculture and Life Sciences, Seoul National University, Seoul, 08802 South Korea; 2 Department of Forestry and Natural Resources, Purdue University, West Lafayette, IN 47906 USA; 3 Department of Landscape Architecture and Rural System Engineening, Seoul National University, Seoul, 08802 South Korea; 4 Warnell School of Forestry & Natural Resources, University of Georgia, Athens, 30602 USA; 5 DMZ Ecology Research Institute, Paju, 10881 South Korea

**Keywords:** Biodiversity, Community ecology, Herpetology

## Abstract

Functional traits are characteristics of species that affect their fitness and ecosystem, and they greatly influence ecological niches. Thus, biodiversity assessment based on functional groups rather than species *per se* can more realistically reflect the ecological niche space. As essential players of ecosystem functions, herpetofauna are appropriate subjects of functional trait-based analyses. In this study, using a nationwide dataset and applying trait information and ecological niche modeling, the richness within each functional group, and the taxonomic and functional diversity indices of South Korean herpetofauna were visualized to identify and compare the geographic distributions. The results revealed that the reptile community seemed more locally diverse with more overlapping randomized patterns among groups than amphibians, while amphibians showed wider distributions and a higher within-grid occurrence ratio. Functional diversity indices of reptiles also showed more randomized geographic patterns with higher levels at Jejudo Island than amphibians. The findings of this study may help to identify biodiversity hot spots and understand its ecosystem health. Increasing survey data and trait information will improve the assessment.

## Introduction

Functional traits can be defined in many ways including any traits that affect morphological, phenological, or physiological processes (e.g., respiration), life-history traits (e.g., growth), traits related to individual fitness or performance measures that can indirectly affect the fitness of an organism^1,2^. Because they can be generalized among similar habitats beyond the species level, functional trait-based indicators are useful tools for assessing the biodiversity of different geographic regions^3,4^. Additionally, using these advanced indicators, the ecological niche configuration of a community can be incorporated into biodiversity assessment^5^. Therefore, the application of functional trait-based indicators is now widely used to explore the responses of biodiversity to ecological disturbance and conservation management^3,6,7^.

A functional group can be defined as a group of species possessing similar functional traits^8^. The richness across all functional groups and the richness within each group of a location determine whether its community is functionally redundant or not, so that whether the community is resilient against species extinction or not^9,10^. Mason *et al*.^11^ firstly proposed three aspects of functional diversity: functional richness, functional evenness, and functional divergence to characterize the function of biodiversity in ecosystems; and Villéger *et al*.^12^ developed multidimensional measurement methods for the three indices. These three aspects describe the area of functional space occupied by the functional group (functional richness), the regularity of species abundance distribution in the space (functional evenness), and the distance of high species abundance from the center of the space (functional divergence). Combining these three aspects, functional diversity may represent the process of species interaction and environmental resource utilization defined by functional traits but also define ecological niche space of the ecological community^13,14^.

Amphibians and reptiles play essential roles for ecosystem processes and services in the environment and society they belong to. For the environment, they aid the nutrient cycling, pollinate and disperse seeds, control pest species, and connect the food web by predating on producers (i.e., plants) or lower-level consumers and being preyed on by higher-level consumers^15–17^. For the society, they serve as a source of foods/medicines for humans and also become a subject in myths, arts, and literature of the traditional culture^15,16^. Therefore, studying the functional diversity pattern of herpetofauna can contribute to understanding the health of ecosystem processes and services of the region.

**Fig. 1:**
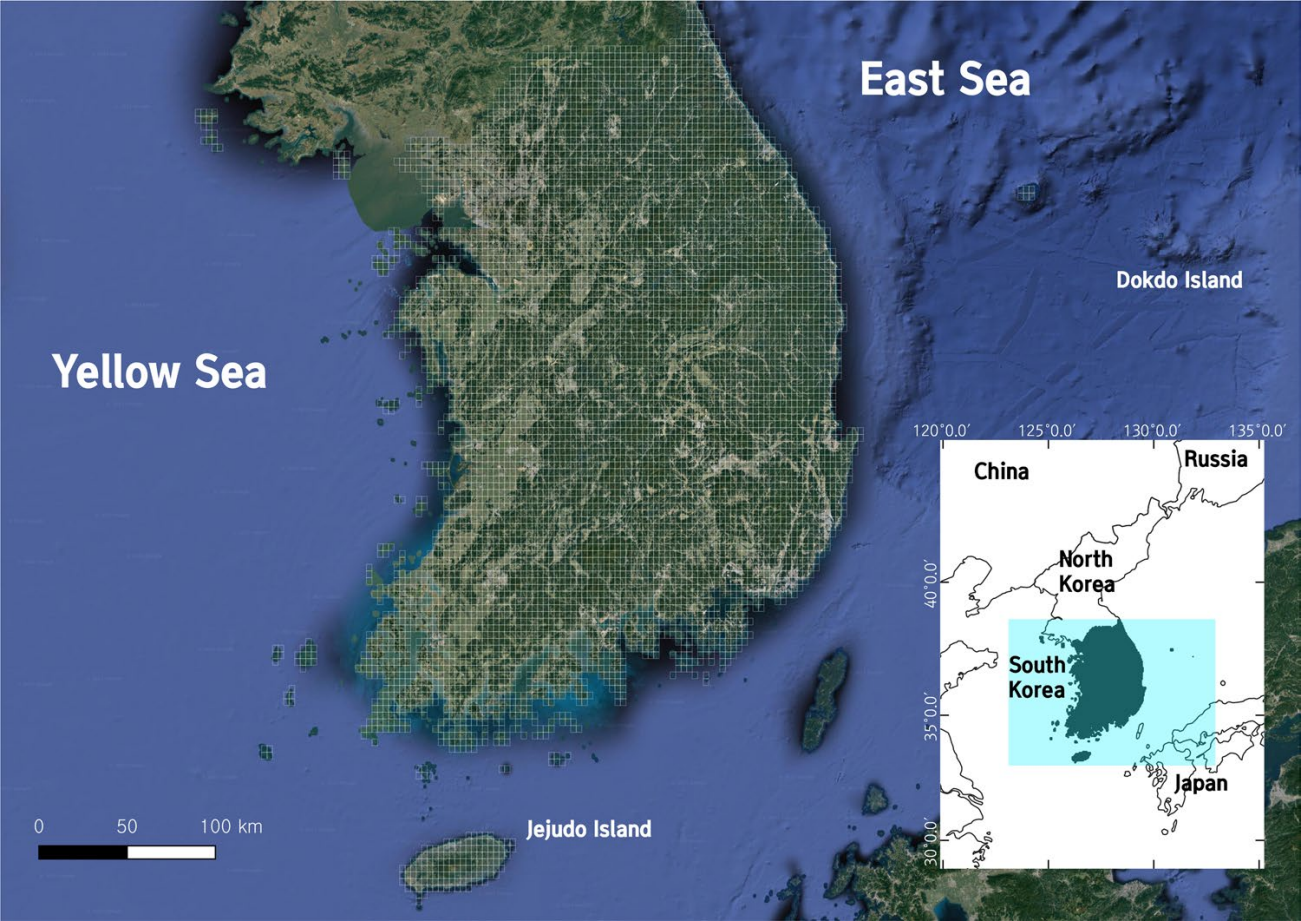
A satellite image of South Korea showing mountainous regions in green (source: *Google Earth*). Survey grids were embedded on the image. The inset shows the location of South Korea in East Asia.

South Korea is located in Northeast Asia between China, Japan, and Russia (Fig. 1). As a glacial refugium for animals during the Pleistocene era^18–20^, its herpetofauna is characterized by unique and cryptic species^21,22^. The Korean Peninsula is home to 32 reptile species and 22 amphibian species (National Institute of Biological Resources, https://species.nibr.go.kr/index.do), and the species list is still growing as more cryptic species are being reported^23–25^, and numerous North Korean species are likely undiscovered^26^. The occurrence of species throughout South Korea has been monitored since 1986 by the National Ecosystem Survey^27^. However, there have been few studies utilizing this nationwide species record, and even fewer have analyzed the biodiversity of this country from this dataset. Against this backdrop, country-scale biodiversity monitoring based on functional traits is beneficial because functional group clustering, above the species level, can account for cryptic species that are not yet described as distinct species but that can be characterized by their traits. If cryptic species are distinguished only by molecular characteristics and their ecological characteristics are undistinguishable from related taxa^22,24^, this approach to biodiversity assessment may be more robust for species that will be described in the future.

In the present study, we explored the distributions of functional groups of amphibians and reptiles in South Korea. We gathered functional trait information for amphibians and reptiles from the literature and various resources (Data File 1 and 2), and clustered functional groups for Order Amphibia and Reptilia according to functional traits. We then exploited the national-scale species occurrence records (Data File 3), and plotted the richness of each functional group, the traditional species richness, and functional diversity indices (functional richness, functional evenness, and functional divergence) on the geographic map of South Korea. Because the functional trait-based analyses incorporate the ecological community composition and reflect ecological niches rather than taxonomic units, we hypothesized that the northern regions of the country will be revealed as functionally diverse, although the southern regions harbor more unique taxons^24,25,28^. This is the first attempt to apply functional traits to assess regional vertebrate biodiversity in Korea, and to characterize the ecological niche structure of amphibians and reptiles in this country on a national scale.

## Results

**Fig. 2:**
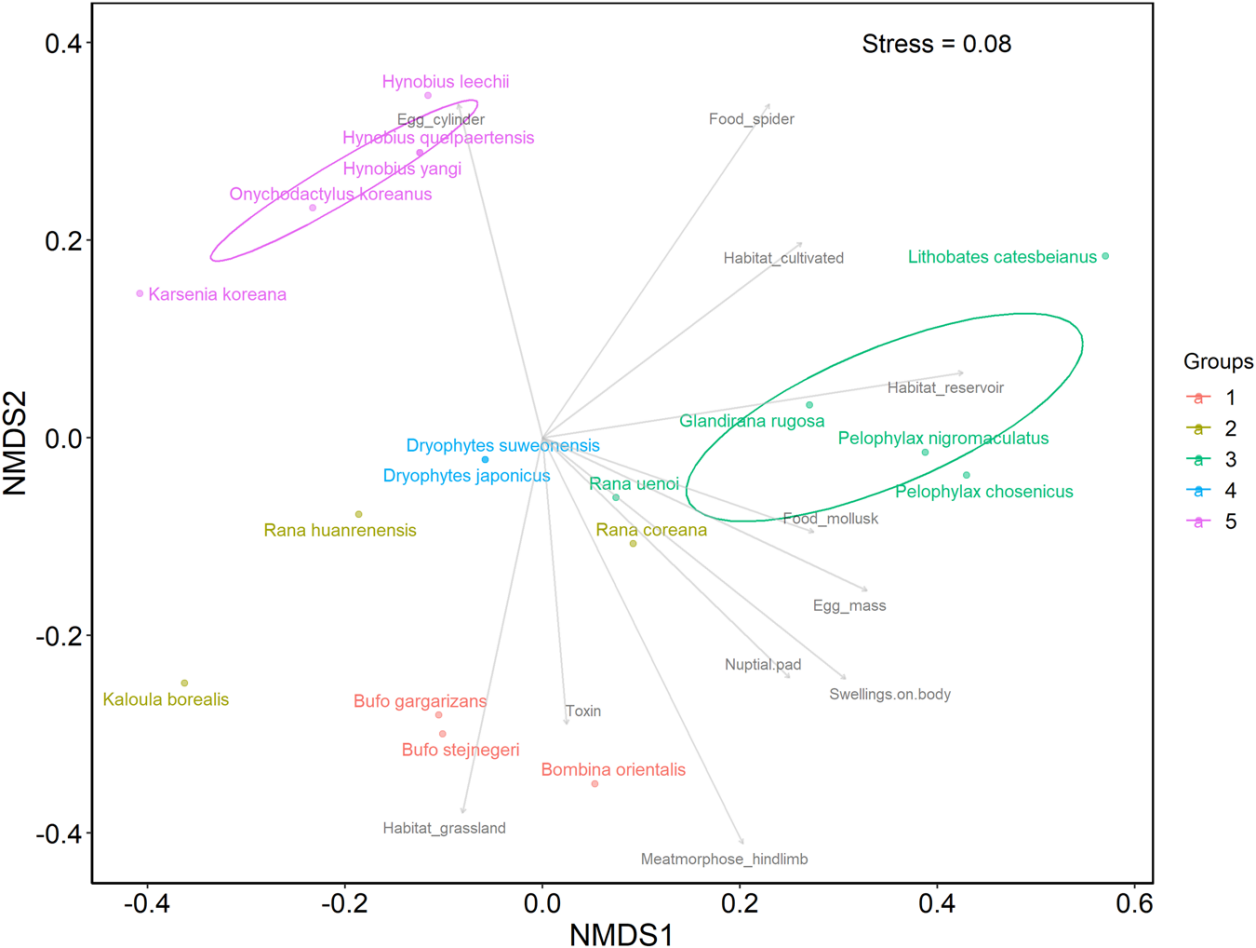
The non-metric multidimensional scaling (NMDS) plot of South Korean amphibians with a stress value of 0.08. Functional groups are color-coded as described in the legend on the right. Statistically significant axes are displayed as gray arrows. Ellipses are drawn only for groups with more than five members.

**Fig. 3:**
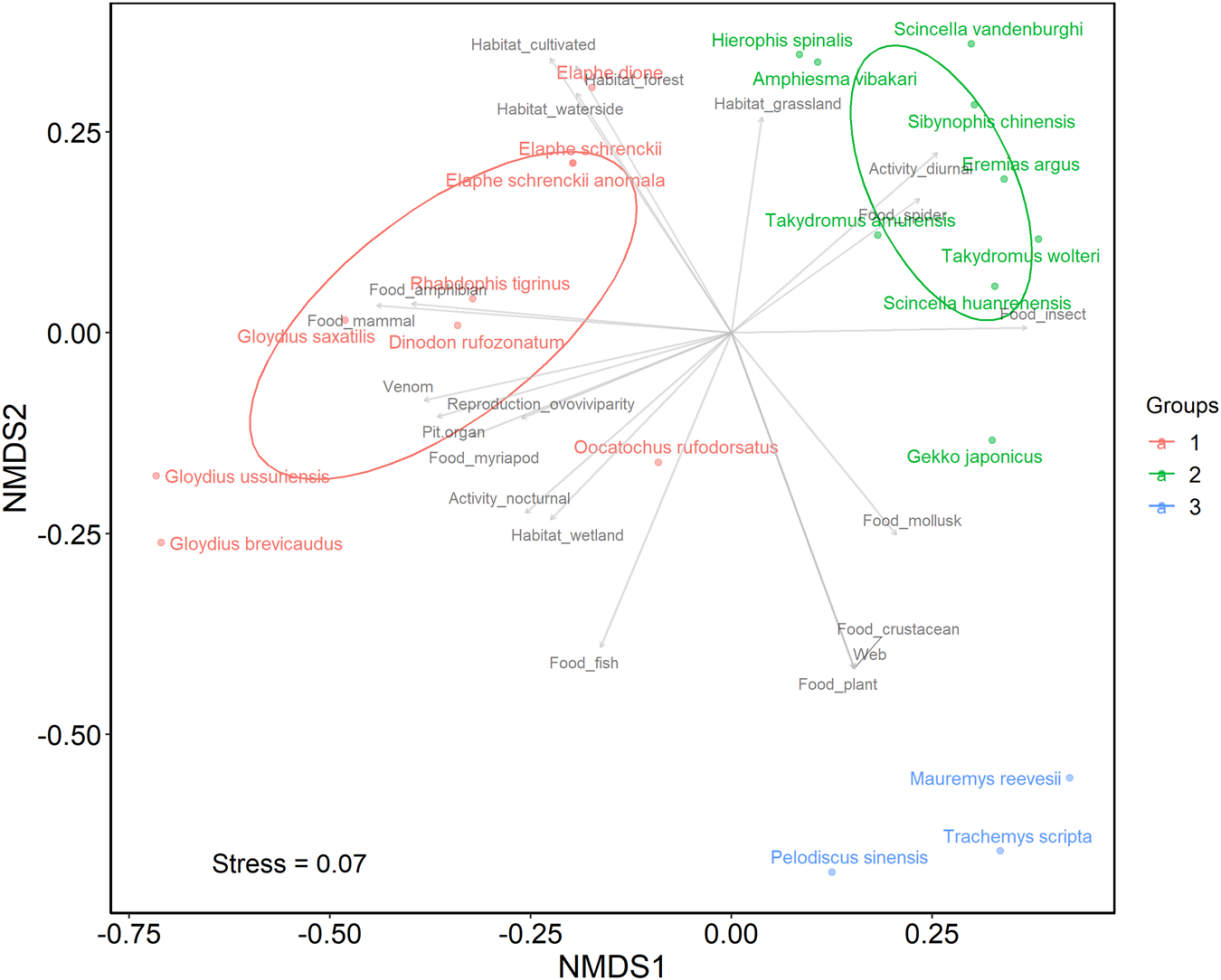
The non-metric multidimensional scaling (NMDS) plot of South Korean reptiles with a stress value of 0.07. Functional groups are color-coded as described in the legend on the right. Statistically significant axes are displayed as gray arrows. Ellipses are drawn only for groups with more than five members.

**Fig. 4:**
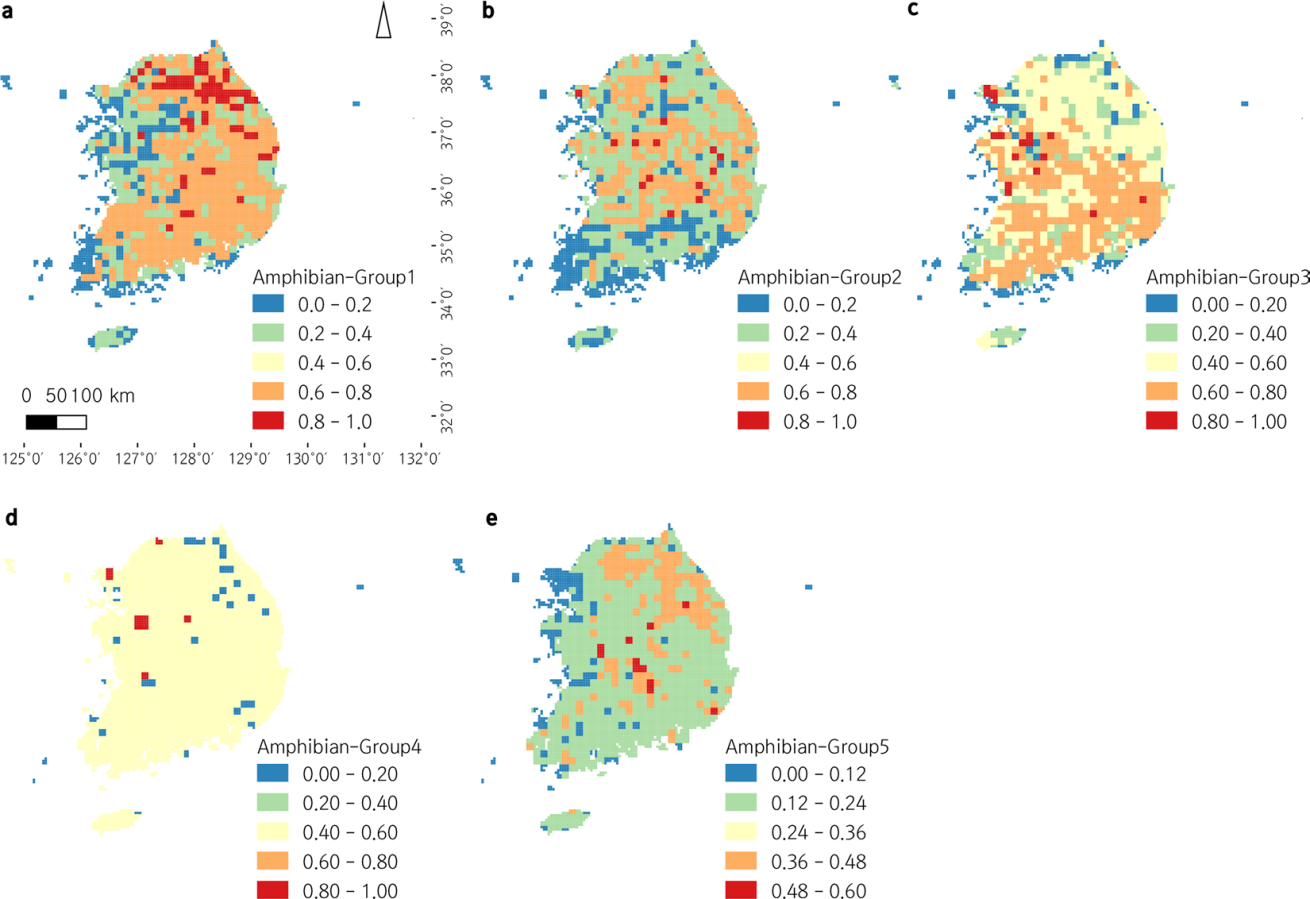
Geographic distribution of amphibian functional groups in South Korea. ‘Within-grid ratios’ (The ratio of recorded species to the number of total species belonging to the functional group within each grid) are color-coded for five levels. (**a**) Functional Group 1 (A1), (**b**) Functional Group 2 (A2), (**c**) Functional Group 3 (A3), (**d**) Functional Group 4 (A4), (**e**) Functional Group5 (A5).

**Fig. 5:**
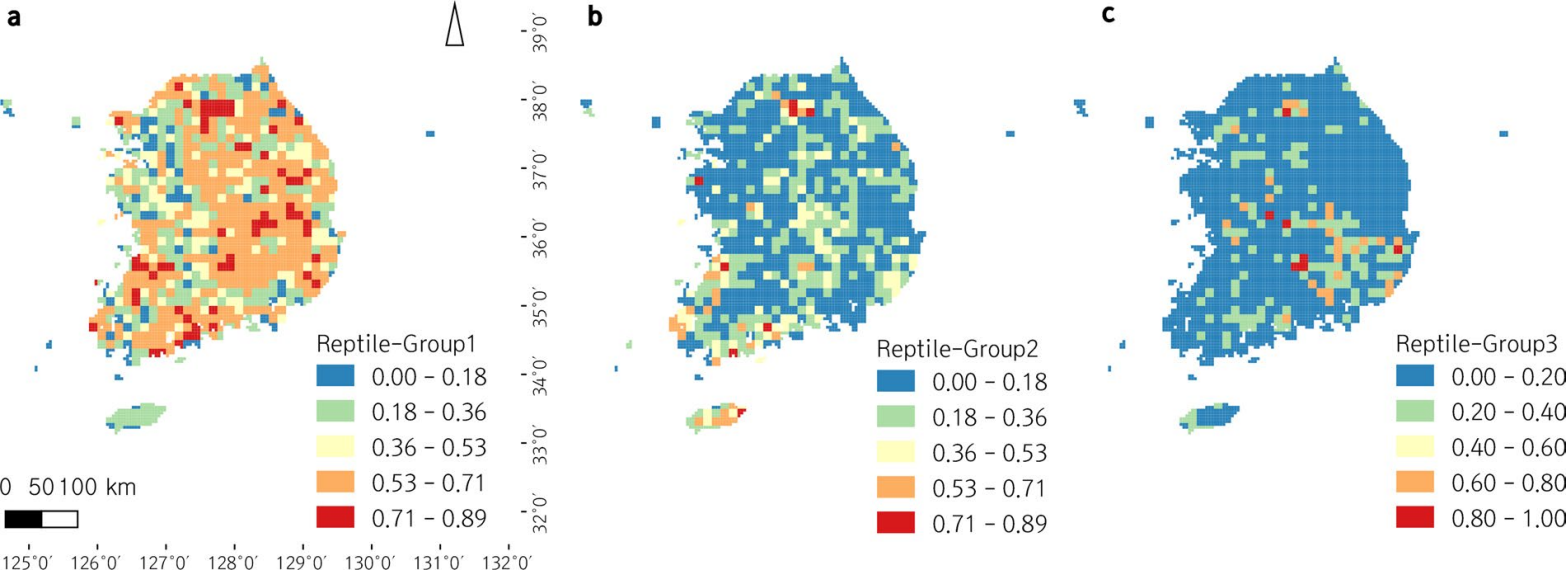
Geographic distribution of reptile functional groups in South Korea. ‘Within-grid ratios’ (The ratio of recorded species to the number of total species belonging to the functional group within each grid) are color-coded for five levels. (**a**) Functional Group 1 (R1), (**b**) Functional Group 2 (R2), (**c**) Functional Group 3 (R3).

**Fig. 6:**
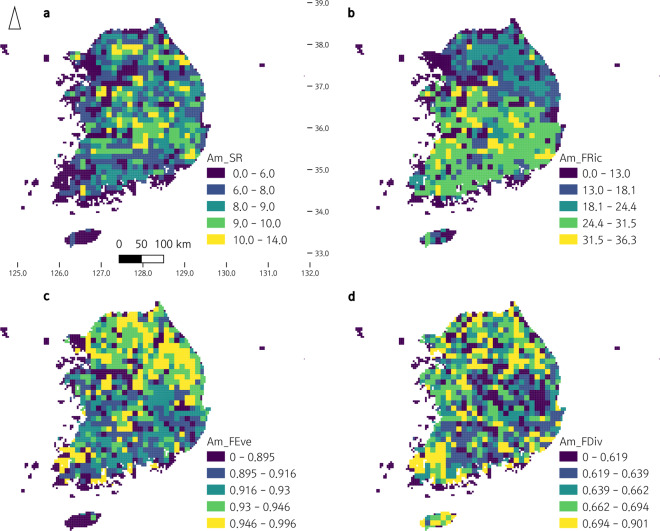
Geographic pattern of amphibian functional diversity indices in South Korea. The distribution of each index was divided into five quantiles and then color-coded. (a) species richness (Am_SR), (b) functional richness (Am_FRic), (c) functional evenness (Am_FEve), (d) functional divergence (Am_FDiv).

**Fig. 7:**
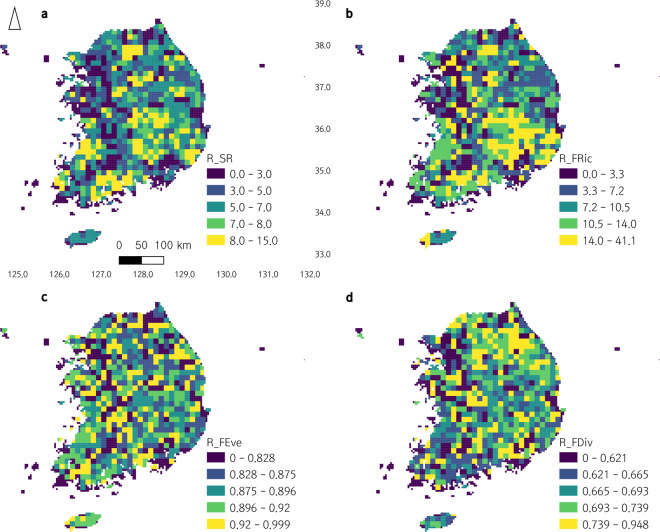
Geographic pattern of reptile functional diversity indices in South Korea. The distribution of each index was divided into five quantiles and then color-coded. (**a**) species richness (R_SR), (**b**) functional richness (R_FRic), (**c**) functional evenness (R_FEve), (**d**) functional divergence (R_FDiv).

Utilizing the 3^rd^ and 4^th^ National Ecosystem Survey data, we clustered South Korean herpetofauna based on functional traits, and analyzed the distributions of functional groups of South Korean amphibians and reptiles in the ecological niche (see METHODS for details; Figs. 2,3). We also visualized the distributions of each functional group along South Korea as a ‘within-grid ratio’ (the ratio of recorded species to the number of total species belonging to each functional group within each grid; Figs. 4,5). Based on the clustered functional groups, we calculated functional richness, functional evenness, and functional divergence, along with the taxonomic species richness of amphibians and reptiles in Figs. 6,7, respectively, after applying Ecological Niche Modeling (ENM; see METHODS for details and see https://hyunkim36.users.earthengine.app/view/enmherptilekor for results including model accuracy). The raw list of species that occurred in each grid was also arranged in Data File 3.

### Ordination analysis and geographic distribution of functional groups

Both non-metric multidimensional scaling (NMDS) plots for amphibians and reptiles indicated good ordination results with stress values lower than 0.1^29^. Amphibian species were clustered into five groups (A1–A5; Fig. 2), and reptile species were clustered into three groups (R1R3; Fig. 3). Their geographic distribution on the map of South Korea were displayed in Figs. 4,5. In general, among amphibian species, all salamanders were well distinguished from frogs based on opposite anuran traits (i.e. ‘Metamorphose_hindlimb’, ‘Toxin’, ‘Nuptial.pad’, ‘Swellings.on.body’, ‘Egg_mass’, ‘Food_mollusk’, ‘Habitat_grassland’, ‘Habitat_reservoir’). Based on the geographic map, Jejudo Island generally displayed fewer species across all amphibian groups. Reptile species were divided into three groups characterized by medium- to large-sized snakes (R1), small snakes and lizards (R2), and freshwater turtles (R3). Mid-northern regions, southwestern regions, and southeastern regions showed higher records for all reptile groups.

Members of group A1 include *Bufo gargarizans*, *B. stejnegeri*, and *Bombina orientalis*, which were explained by ‘Toxin’ and ‘Habitat_grassland.’ They are present in northeastern and mid-southern regions of South Korea. The A2 group, which includes *Kaloula borealis*, *Rana huanrenensis*, and *R. coreana*, is the least peculiar amphibian group in terms of its distribution. No specific explanatory variable was found on the NMDS plot and no particular pattern was observed on the map. However, the A2 numbers were relatively infrequent along with mainland coastal areas. The A3 group consists of *Pelophylax nigromaculatus*, *P. chosenicus*, *Glandirana rugosa*, *Rana uenoi*, and *Lithobates catesbeianus*, the invasive species, which was distantly located from others. NMDS axes ‘Habiat_reservoir’, ‘Habitat_cultivated’, ‘Food_mollusk’, ‘Food_spider’, ‘Egg_mass’, ‘Nuptial.pad’, and ‘Swellings.on.body’ primarily explained this group. On the geographic map, this group is more common in the southern half of South Korea. The genus *Dryophytes* was only included in group A4, and two species (*D. japonicas* and *D. suweonensis*) were located at the same spot on the NMDS plot. Unlike other groups, the highest occurrence on the map is concentrated among specific regions, reflecting the confined distribution of *D. suweonensis*^30^. Group A5 (salamanders) includes all salamander species. As mentioned above, this group could be explained by the egg shape trait and the absence of anuran traits on the opposite side of the NMDS plot. On the map (Fig. 4), since *Hynobius leechii* is distributed throughout South Korea, areas where other species are co-occurring with this species (southeastern coastal area - with *H. yangi*, southwestern regions - with *H. quelpaertensis*, northeastern and middle regions - with *Karsenia koreana* and *Onychodactylus koreanus*) had a higher frequency of group A5^24^.

R1 consists of *Elaphe dione*, *E. schrenckii*, *E. schrenckii anomala*, *Oocatochus rufodorsata*, *Dinodon rufozonatum*, *Rhabdophis tigrinus*, *Gloydius ussuriensis*, *G. brevicaudus*, and *G. saxatillis*, making it the most widespread group. *O. rufodorsata*, is relatively distant from the center of the group. This group could be explained by the majority of trait axes on the NMDS plot, including diverse habitat types (‘Habitat_forest’, ‘Habitat_cultivated’, ‘Habitat_waterside’, ‘Habitat_wetland’), food types (‘Food_amphibian’, ‘Food_mammal’, ‘Food_myriapod’, ‘Food_fish’), traits of the genus *Gloydius* (‘Venom’, ‘Pit.organ’, ‘Reproduction_ovoviviparity’), and ‘Activity_nocturnal.’ The ‘Activity_diurnal’, ‘Food_spider’, ‘Food_insect’, ‘Food_mollusk’, ‘Food_crustacean’, ‘Food_plant’, and ‘Web’ axes were at the opposite end of the cluster. This group occurs throughout the country on the geographic map, while Jejudo Island and the region near the capital Seoul displayed lower frequencies than other regions. The R2 group members include *Scincella vandenburghi*, *S. huanrenensis*, *Takydromous amurensis*, *T. wolteri*, *Eremias argus*, *Gekko japonicas*, *Amphiesma vibakari*, *Sibynophis chinensis*, and *Hierophis spinalis*. *Gekko japonicas* stands alone from other members. ‘Activity_diurnal’, ‘Food_spider’, and ‘Food_insect’ are the most significant axes explaining this group, while the most significant axes explaining R1 are at the opposite end of the cluster. The number of species in this group recorded in each grid on the geographic map was relatively low across the country, except in mid-northern regions, southwestern regions, and Jejudo Island. The R3 group includes *Pelodiscus sinensis*, *Mauremys reevesii*, and the invasive species, *Trachemys scripta*. Traits related to underwater life, including ‘Web’, ‘Food_mollusk’ (e.g., freshwater bivalves), ‘Food_crustacean’, and ‘Food_plant’ contributed to clustering these freshwater turtles. ‘Habitat_forest’, ‘Habitat_cultivated’, and ‘Habitat_waterside’ were at the opposite end of the cluster for this group. The occurrence showed dispersed pattern, excluding southeastern regions with higher occurrence, on the geographic map.

### Species richness and functional diversity calculation and their relationship with topographic characteristic in South Korea

Species richness (SR) and functional diversity indices (functional richness – FRic, functional evenness – FEve, and functional divergence – FDiv) were calculated and visualized on a map of South Korea for amphibians and reptiles (Figs. 6,7; Data File 4), respectively. For amphibians, SR ranged 0–14 (mean: 8.12, SD:2.02), FRic ranged 0.0–36.3 (mean: 21.502, SD:8.898), FEve ranged 0.000–0.996 (mean: 0.922, SD: 0.033), and FDiv ranged 0.000–0.901 (mean: 0.662, SD: 0.050). For reptiles, SR ranged 0–15 (mean: 6.547, SD: 2.249), FRic ranged 0.0–41.1 (mean: 13.034, SD: 10.350), FEve ranged 0.000–0.999 (mean: 0.890, SD: 0.052), and FDiv ranged 0.000–0.948 (mean: 0.700, SD: 0.063).

The geographic pattern of these indices was not generally concordant each other, while FRic of reptiles followed the pattern of its SR overall (Figs. 6,7). Amphibians were identified having high SR all over the country except the west coast, south coast, and Jejudo Island. Compared to SR, FRic was higher in southwestern and southeastern regions and lower in northern and northeastern regions of the country. FEve showed the opposite pattern of FRic, displaying higher levels mostly along the middle, northern, and northeastern regions of the country. FDiv levels were generally high all over the country including Jejudo Island, except in several regions including southeastern regions of relatively lower levels.

Reptiles had mostly high levels of SR throughout the mainland except western regions where the capital Seoul and some other large cities located, showing lower levels. FRic displayed a similar pattern of SR and indicated the highest levels in southeastern regions. Mid-northern and middle regions were identified having high levels for both SR and FRic. Levels of FEve and FDiv were more homogeneous than SR and FRic. Not a particular pattern was found for both. Still, consistently for all four indices, regions near Seoul had lower levels. Jejudo Island had relatively high levels of FRic and FEve.

**Fig. 8:**
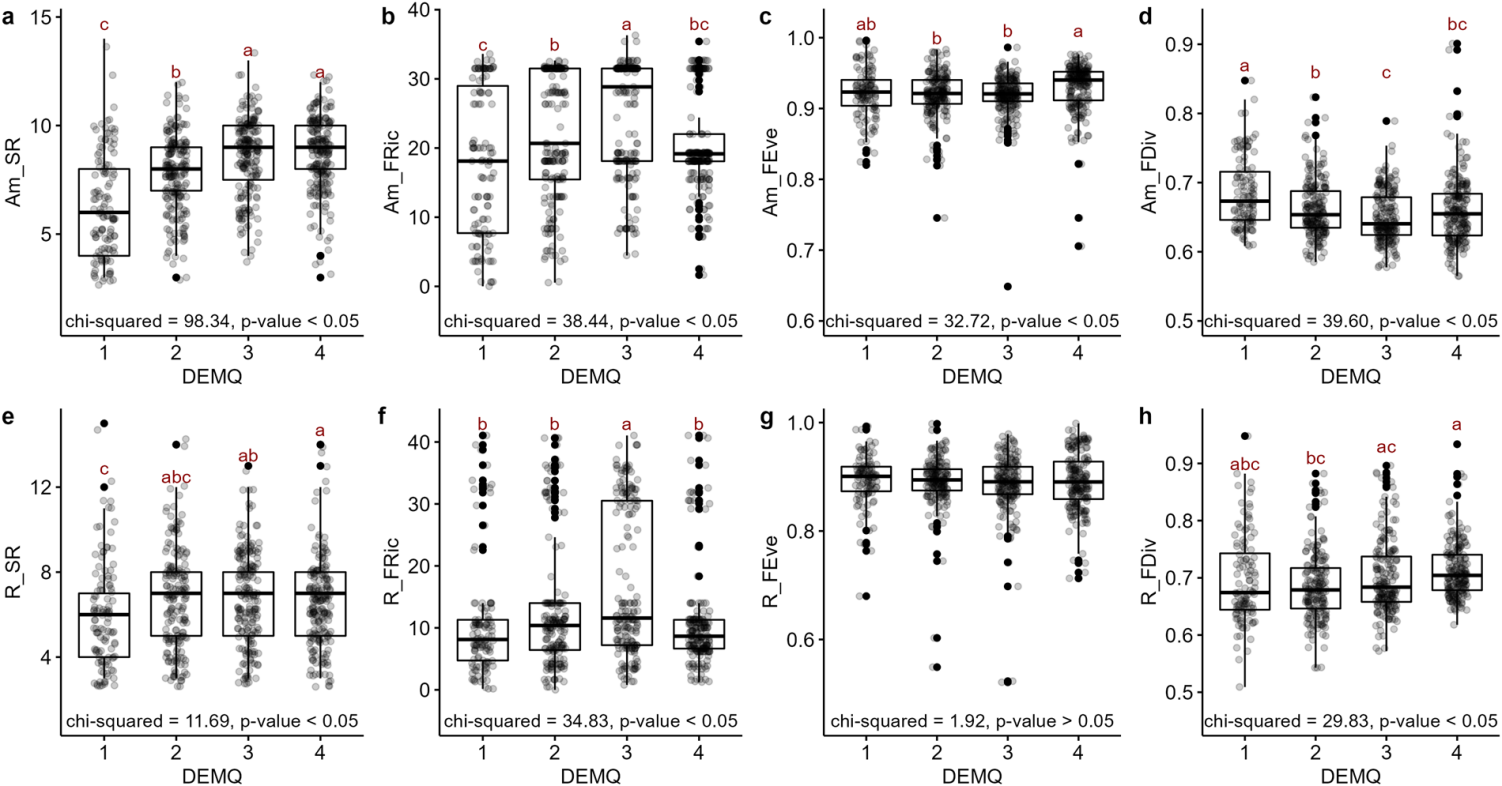
The relationship between DEM and herpetofauna diversity indices. DEMQ stands for the DEM quartile (DEMQ. 1st: 0.113–15.855 m, DEMQ. 2nd: 15.855–72.325 m, DEMQ. 3rd: 72.325–247.675 m, DEMQ. 4th: 247.675–987.383 m). Amphibian diversity and elevation: (**a**) species richness (Am_SR), (**b**) functional richness (Am_FRic), (**c**) functional evenness (Am_FEve), (**d**) functional divergence (Am_FDiv). Reptile diversity and elevation: (**e**) species richness (R_SR), (**f**) functional richness (R_FRic), (**g**) functional evenness (R_FEve), (**h**) functional divergence (R_FDiv). Groups sharing the same non-capital letters did not show statistically significant differences in Kruskal-Wallis test with Games-Howell post-hoc test.

**Fig. 9:**
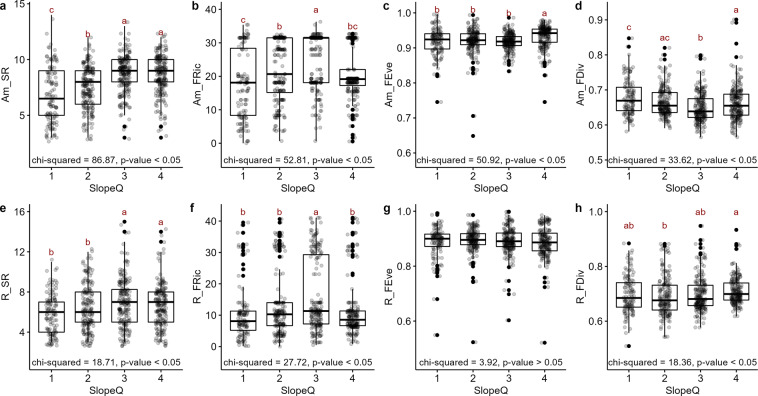
The relationship between slope and herpetofauna diversity indices. SlopeQ stands for the slope quartile (SlopeQ. 1st: 0.115–1.973°, SlopeQ. 2nd: 1.973–6.727°, SlopeQ. 3rd: 6.727–12.227°, SlopeQ. 4th: 12.227–19.523°). Amphibian diversity and slope: (**a**) species richness (Am_SR), (**b**) functional richness (Am_FRic), (**c**) functional evenness (Am_FEve), (**d**) functional divergence (Am_FDiv). Reptile diversity and slope: (**e**) species richness (R_SR), (**f**) functional richness (R_FRic), (**g**) functional evenness (R_FEve), (**h**) functional divergence (R_FDiv). Groups sharing the same non-capital letters did not show statistically significant differences in Kruskal-Wallis test with Games-Howell post-hoc test.

Regarding the relationship of biodiversity indices with topographic characteristics (Figs. 8, 9), the SR and FEve of amphibians were the highest in DEMQ. 4, the highest elevation range (Kruskall-Wallis test, *p* < 0.05). FRic of amphibians was the highest in DEMQ. 3, the mid-high elevation range (Kruskall-Wallis test, *p* < 0.05). FDiv of amphibians was the highest in DEMQ. 1, the lowest elevation range (Kruskall-Wallis test, *p* < 0.05). The SR and FDiv of reptiles were the highest in DEMQ. 4 (Kruskall-Wallis test, *p* < 0.05). FRic of reptiles was the highest in DEMQ. 3 (Kruskal-Wallis test, *p* < 0.05). FEve of reptiles was no significant difference between elevation levels.

The SR, FEve and FDiv of amphibians were the highest in SlopeQ. 4, the steepest mountain area (Kruskall-Wallis test*, p* < 0.05). FRic of amphibians was the highest in SlopeQ. 3, the mid-to-steep range (Kruskall-Wallis test*, p* < 0.05). SR of reptiles was the lowest in SlopeQ. 1, the flatland where most developed cities are located (Kruskall-Wallis test*, p* < 0.05). FRic of reptiles was the highest in SlopeQ. 3 (Kruskall-Wallis test*, p* < 0.05). FDiv of reptiles was the lowest in SlopeQ. 2 (Kruskall-Wallis test*, p* < 0.05), the mid-to-flat range. FEve of reptiles was no significant difference between slope ranges.

## Discussion

In this study, we identified the distributional patterns of functional groups and functional diversity of South Korean herpetofauna using diverse ecological and morphological traits from various information sources, such as databases and field guides, and large-scale species occurrence data from National Ecosystem Survey conducted throughout the country from 2006 to 2018. In general, the results were well matched with our hypothesis of the northern regions as functionally more diverse than the southern counterparts. We identified the northern and also the eastern regions of South Korea, where large mountains are located (Fig. 1), have generally higher values of diversity indices. This was because the cryptic species with similar traits that share ecological niches were grouped into the same functional groups, reflecting ecologically functional units into downstream analyses rather than taxonomic units. Therefore, the northern and eastern regions, which harbors relatively higher ratios for most of herpetofauna functional groups than other regions, showed higher functional diversity (i.e., FEve and FDiv) on the contrary to taxonomic diversity (i.e., SR). Indeed, the result is plausible in that the large mountain chain, so-called “Backdudaegan”, stretched from the northeastern regions has already found as a major biodiversity hotspot of the Korean Peninsula^31^.

Looking into the results further, functional groups and their distributions effectively reflected the ecological characteristics of group members on the NMDS plot and the map of the country. A3 and R1 groups were the most dominant groups on the NMDS and geographic map for each amphibians or reptiles with the largest number of members and generally high occurrence along the country. Wide habitat and prey spectrum, relatively high dispersal ability, and prolific reproducibility (especially the invasive *Lithobates catesbeianus* in group A3) of the members in those groups^32–34^ seemed contribute to the dominant distribution.

Order Amphibia included more functional groups than Order Reptilia, even though it has fewer species overall. Functional groups of amphibians were divided into anurans and urodeles at a glance (Fig. 2), and this might be because of different habitat preferences and egg morphology between salamanders and frogs^33^. Additionally, the maximum ratio of species occurrence at a given grid point (i.e., maximum ‘within-grid ratio’; see legends of Figs. 4,5) and the general level of species occurrence throughout the geographic area were typically higher among amphibian groups than reptile groups, which implied amphibians are functionally more redundant than reptiles so that more resistant to local extinction of species^35^. The wider distribution and higher abundance of amphibians could explain these observations. Distributions of functional groups seemed to mostly reflect the habitat preference of the group’s members (Data File 1), for detailed examples, A1 - favoring forests, grasslands, and streams; A2 - avoiding large river habitats^32,36^; A3 - favoring lowland areas rather than highland areas in northern regions^32,37^; and A5 (*Karsenia koreana* and *Onychodactylus koreanus*) - favoring mountainous areas (northeastern and middle regions)^33^.

There were some noteworthy cases from results, such that an invasive member of A3, *Lithobates catesbeianus*, was distant from other members within the group on the NMDS plot. This would be somewhat relieving from the viewpoint of conservation, since the result implies that this invasive species occupies a non-overlapping ecological niche from its functional group’s native amphibian members, which might lessen interspecific competitions and further imply species-specific management measures later. Still, as the species might have other negative effects in the ecosystem, such as voracious predation of native species, interpretation needs caution. The distant location of this invasive species by the NMDS is possibly due to more diverse prey items of this invasive species than the other native members of the group, as evidenced by ‘Food_amphibian’, ‘Food_fish’, and ‘Food_crustacean’ (Data File 1), but these were not significantly represented in the plot. Although not shown on the plot either, group A4 seemed differentiated from other groups by the ‘Toe.pad’ trait (Data File 1).

The reptile NMDS plot clustered species well according to their functional traits. The majority of trait axes accounted for the group R1 and this reflected the general habitat preferences and food resources of this group, especially the most bulky food item (i.e., mammals) exclusive to this group (Data File 2). Significant trait axes explaining R2 were related to behavioral characteristics of its members (i.e., basking in the sun of lizards) and the preference for small prey items^32^.

Geographic distributions of reptile functional groups largely overlapped in the southeast and southwest regions of the country, compared to amphibian groups that were more separated from each other. These results imply that reptile communities are functionally more diverse at the local level. In detail, R1 commonly occurred across mainland South Korea which is consistent with the wide range of the group’s habitat preferences. However, it was not well represented in Jejudo Island and along western metropolitan areas because islands are expected to have fewer species^38^ and regions surrounding Seoul and large cities lack appropriate habitats. In contrast, R2 recorded more members in Jejudo Island than any others including amphibian groups, probably since the island has unique species (i.e., *Sibynophis chinensis*) and a large population of *Takydromus wolteri* in addition to other members^38^. Moreover, this group showed a higher number of members in some southwestern regions, as the regions have a larger population of *Scincella vandenburghi*, as well as northern regions of the country in which *Scincella huanrenensis* is found exclusively^38^. Having members mostly among southeastern regions, R3 had no members in mountainous areas of the country because turtles prefer large waterbodies and lowland areas.

Two notable outlier reptile species from the results were *Oocatochus rufodorsata* and *Gekko japonicas*. *Oocatochus rufodorsata* was relatively distant from the center of the group on the NMDS plot due to its distinct preference for shrub, wetland, and waterside habitats, but not forest, unlike other members in its group^32^ (Data File 2). *Gekko japonicas* also stood alone from other members of R2, distinguished by its peculiar characteristics such as favoring developed areas for the primary habitat and nocturnal activity (Data File 2). The invasive species of reptiles, *Trachemys scripta*, did not show noticeable difference from other members of its group.

Generally, for both amphibians and reptiles, functional diversity indices had geographic patterns (Figs. 1, 6–9). FRic mirrored the geographic pattern of SR^14^, while the geographic patterns of FEve and FDiv were more similar each other, which are patterns commonly found in the previous studies^35,39,40^. SR was lower especially at the lowest elevation and slope and this was reflected as relatively low SR values along the western regions where large cities located. It could be due to low primary productivity and relative habitat homogeneity of flatland^39,40^. FRic were relatively low along the northeastern regions, the highest and steepest mountainous area of the country. The cold temperature of this area of too high elevation and slope might lower herpetofauna functional diversity^39^, considering their temperature-dependence as ectotherms. In contrast, FEve and FDiv were generally higher in the northern and northeastern regions of the highest elevation and slope, implying potentially lower intensities of competition and invasion^11,14^ among diverse habitats in the mountainous area^39^. Reptile FEve and FDiv were relatively homogenized than amphibians throughout the country but showed the pattern of high values along the western and southern regions where SR and FRic were low. For some reasons, species therein might utilize the entire ecological niche evenly and be resistant against to invasive species^11,14^, although those regions include smaller number of species and volume of functional trait space. The reason of this is remained to be answered for future research.

As a whole, regarding the ecological processes and services the herpetofauna provide, the northern and eastern regions of the country with mountainous terrain have healthier and more sustainable ecosystems than the other regions, especially the western regions with large cities. This can be indirectly proved when the regions of high FEve and FDiv (Fig. 6) are compared with the distribution of A3 (Fig. 4) that includes an invasive species, *Lithobates catesbeianus* (also directly with the occurrence distribution of this species; Data file 3). The healthier northern and eastern regions with high FEve and FDiv values might ward off the spread of the invasive species, although it can easily occupy mountainous habitats^41,42^. The ecosystems of the southern and western regions, on the other hand, are relatively less functional except some restricted regions along the south and west coast (including Jejudo Island) harboring a few unique species. Cautions are need for the growing amount of herpetofauna import^43^ through the coasts. Since many studies revealed that herpetofauna of the Korean Peninsula are especially susceptible to climate change^28,44–46^, the southern ecosystems need a particular conservation concern as habitats there will be disappeared sooner than later^45–48^.

A caveat of the study would be the limited availability of traits at the moment, considering the number of the functional group defined is the determinant of functional group richness pattern^49^ and different traits result in different functional groups^49–51^. In particular, we could not use body size as a variable because 1) it was the only continuous variable with wide spectrum (all the other variables could be binarized) so it functioned as a discriminating factor masking effects of other variables in all the analyses, and 2) body size estimates of some species were not consistent among different sources so there was no one reliable reference to follow. Still, we advocate using body size in these types of analysis whenever possible as it has been reported to affect species’ geographic distribution and ecological niche^52,53^.

We believe that the functional diversity measurement of South Korean herpetofauna will be more robust and improved in the future with consistent basic ecological studies for species, accumulating survey data, and increasing data availability in herpetofauna functional trait databases, such as AmphiBIO^54^. The findings will contribute to the understanding of the functional diversity of vertebrates in South Korea^4^. This study would be an imitable example of using spatio-temporally large-scale survey data to assess the nationwide biodiversity.

## Methods

### Data collection

We used occurrence data for amphibians and reptiles throughout South Korea recorded during the 3^rd^ and 4^th^ National Ecosystem Surveys (the data can be found at: https://www.nie-ecobank.kr/rdm/rsrchdoi/selectRsrchDtaListVw.do or Data File 3). These surveys were conducted from 2006 to 2018 by entrusted herpetologists in groups of two. Following the official survey guideline^27^, the surveys covered all over the national territory which was divided into 824 grids of 1:25,000 scale. Each 1:25,000 topographic grid was then subsequently divided into nine finer-grids of 2′30″ × 2′30″ (~4.5 km) along the latitude and longitude. The teams of herpetologists repetitively surveyed one site per finer-grid, 1–3 times throughout a year, and recorded species occurrences for each individual survey, which pooled into one large data of the year by the managing institution at the end. Although we assumed that the surveys were conducted in a consistent manner following the guideline, we did not use records of species abundance but only used presence/absence from the data because the abundance of each species could be biased due to potentially different sampling efforts, selection criteria of survey sites, and proficiency among investigators. Rather we used the “ENM value” (i.e., the predicted suitability value) in place of species abundance (see section ‘Species richness and functional diversity calculation’ below for details of ENM).

The data contained taxonomic information (e.g., scientific name and common names), locality information (e.g., survey grid numbers and geographic coordinates), and survey date. Combining the data of National Ecosystem Surveys yielded two classes, four orders, 15 families, 25 genera, and 42 species (including one subspecies three non-native turtle species), and 63,303 occurrence records (Supplementary Table 1, Data File 3). We also calculated and added the percentage of area each species recorded (‘% of area’) in grid units in Supplementary Table 1. In the 4^th^ survey data, eight records data of three non-native turtle species were excluded for analyses later. The official data were provided by the National Institute of Ecology in South Korea. We followed the 2020 National Species List of the National Institute of Biological Resources for Korea (https://species.nibr.go.kr/index.do) for scientific names and original data sources for subspecies names when applicable, due to taxonomic equivocacy among some of the local species within the Korean Peninsula (e.g., Jeon *et al*.^55^; Othman *et al*.^56^) and the availability of official species list at the time of study. Two non-native, invasive species on the National Species List, *Lithobates catesbeianus* and *Trachemys scripta*, were also included in the study for comparison with other native amphibians and reptiles, respectively.

Functional trait data for species were collected from published field guidebooks, encyclopedias for South Korean amphibians and reptiles^32–34^, the official biodiversity database managed by the Korean government (National Biodiversity Center, https://www.kbr.go.kr/index.do), and AmphibiaWeb (https://amphibiaweb.org/). We collated morphological traits, habitat preferences, and feeding and behavioral characteristics, and transformed them into binary data (except ‘vocal.sac’ for amphibian traits, which ranged from 0−2). The detailed categories and the rationale or references for usage of these traits were summarized in Table 1. The data used in downstream analyses have been submitted as Data File 1 and 2. Two *Pelodiscus* species (*P. sinensis* and *P. maackii*) in the survey data were treated as one species, *Pelodiscus sinensis*, for analyses due to information unavailability for *P. maackii* in published field guidebooks^32,34^ and historical taxonomic equivocacy between them^57^.

**Table 1:** Functional traits used in this study for each amphibian and reptile species. Mutually exclusive and non-exclusive categories were separated by “/” and “, ”, respectively.

Class	Trait	Categories	Rationale or reference
Amphibia	Vocal sacs	count	Anuran vocal sacs are indispensable tools for their communication^61^.
	Swellings on the body	presence/absence	Barnett (2013)^62^
	Nuptial pad	presence/absence	Liu (1936)^63^
	Toe pad	presence/absence	Li *et al*.^64^
	Egg shape	mass/string/cylindrical/oval	The ovipositional mode and structure of amphibian eggs are the evolutionary consequence of ecological factors, such as predation and environmental conditions^65^.
	Habitat types	forest, shrub, grassland, wetland, stream, river, reservoir, cultivated,	Tsianou *et al*.^49^
	Food items	mollusk, crustacean, annelid, spider, insect, fish, amphibian	Tsianou *et al*.^49^
	Activity	diurnal, norturnal	Tsianou *et al*.^49^
	Toxin	presence/absence	Lourenço‐de‐Moraes *et al*.^51^
	Metamorphosis	development order of hindlimbs/forelimbs	Amphibians that develop forelimbs first during the metamorphosis (i.e., salamanders) can control pest species early on even at the larval stage^66^ but the others that develop hindlimbs first (i.e., frogs) cannot because their tadpoles are not carnivorous.
Reptilia	Keels on scale or on shell	presence/absence	Keels can further protect scales^67^ and improve structural integrity of shells while reducing predation risk^68^.
	Web	presence/absence	Webs on feet can facilitate swimming in the water^69^.
	Pit organ	presence/absence	Pit organ is an important sensory system that detects ambient signals including infrared radiation and temperature^70^.
	Habitat types	forest, shrub, grassland, wetland, waterside, waterbody, cultivated, developed	Santos & Cheylan (2013)^71^
	Food items	plant, mollusk, crustacean, annelid, myriapod, spider, insect, fish, amphibian, reptile, bird, mammal	Santos *et al*. (2013)^71^
	Activity	diurnal, nocturnal	Carvajal-Cogollo *et al*. (2015)^72^
	Venom	presence/absence	Jackson *et al*. (2019)^73^
	Reproductive mode	oviparity/ovoviviparity	Carvajal-Cogollo *et al*. (2015)^72^

### Ordination analysis of species functional traits

To examine ecological divergence within South Korean amphibian and reptile communities from National Ecosystem Survey data, we first separately clustered amphibians and reptiles according to previously collected functional traits (Data File 1 and 2) using the R^58^ package NbClust^59^ in Rstudio. We used the default ‘Euclidean distance’ dissimilarity matrix, ‘ward.D2’ method, and the ‘cindex’ index for clustering because these options can help identify species of minimal within-cluster variance and also the result clusters were well matched with their taxonomy. Clustered species trait data were then subjected to non-parametric multidimensional scaling (NMDS) using the R package vegan^60^. We used ‘Gower’ distance for NMDS analysis that can take many different types of inputs, such as continuous, binary categorical, and non-binary categorical. NMDS plots were separately generated for amphibians and reptiles, and only significant functional trait variables were drawn as arrows in plots. Stress values were calculated.

### The geographic distribution of functional groups in South Korea

We downloaded 1:25,000 maps from the Geospatial Information Platform, a public web service run by the National Geographic Information Institute of Korea (http://map.ngii.go.kr/ms/map/NlipMap.do), for the entire geographic range of South Korea. Each map was used as one grid of a 7′30″ × 7′30″ (approximately 13.5 km × 11.1 km) matrix. The list of species identified from the 3^rd^ and 4^th^ National Ecosystem Surveys was organized and converted into binary data (presence and absence) within each survey grid.

**Fig. 10:**
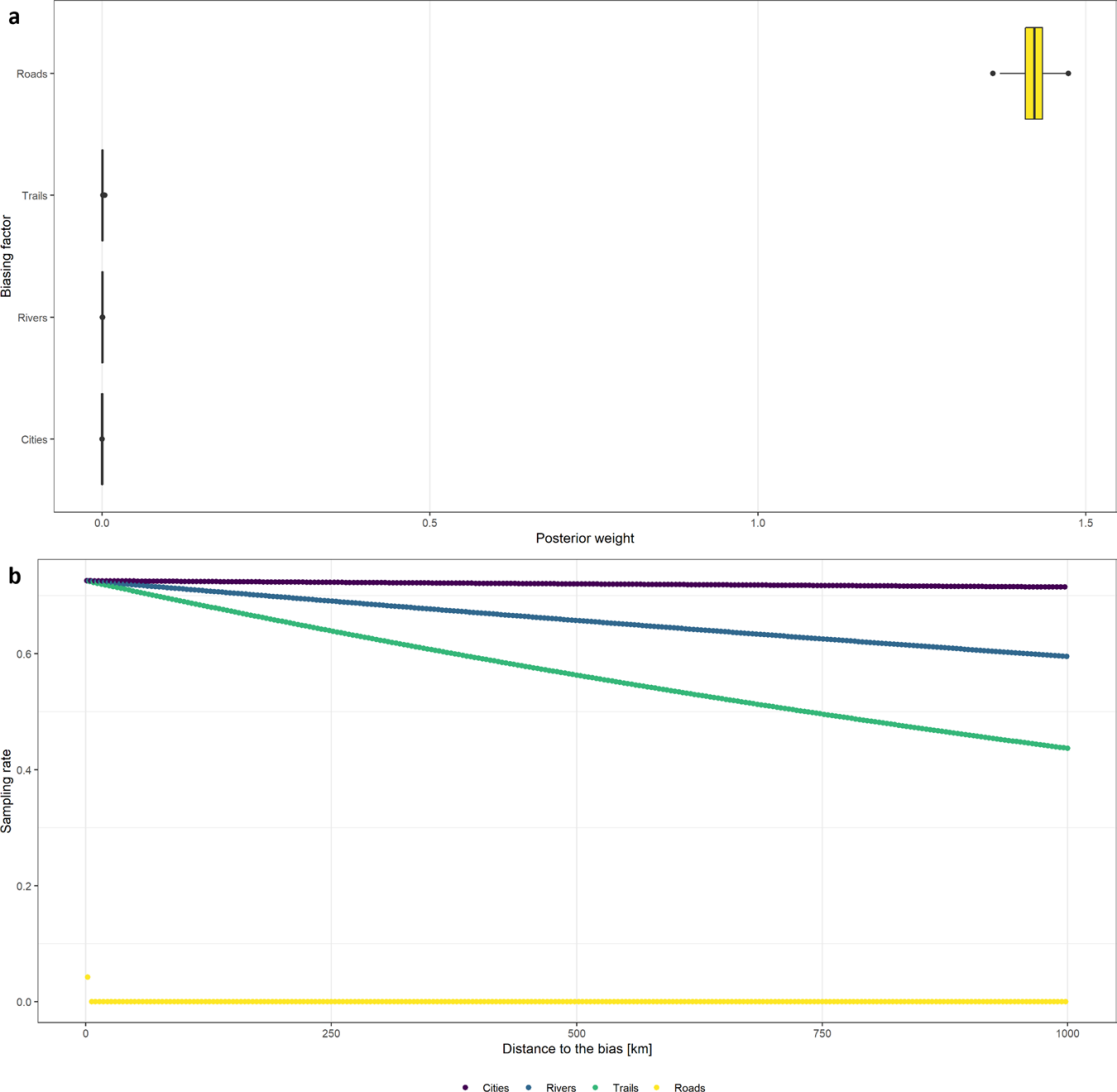
The result of potential sampling bias analysis from roads, trails, rivers and cities, showing the posterior bias weights of each biasing factor (**a**) and the sampling rate according to the increased distance to the biasing factor (**b**).

**Fig. 11:**
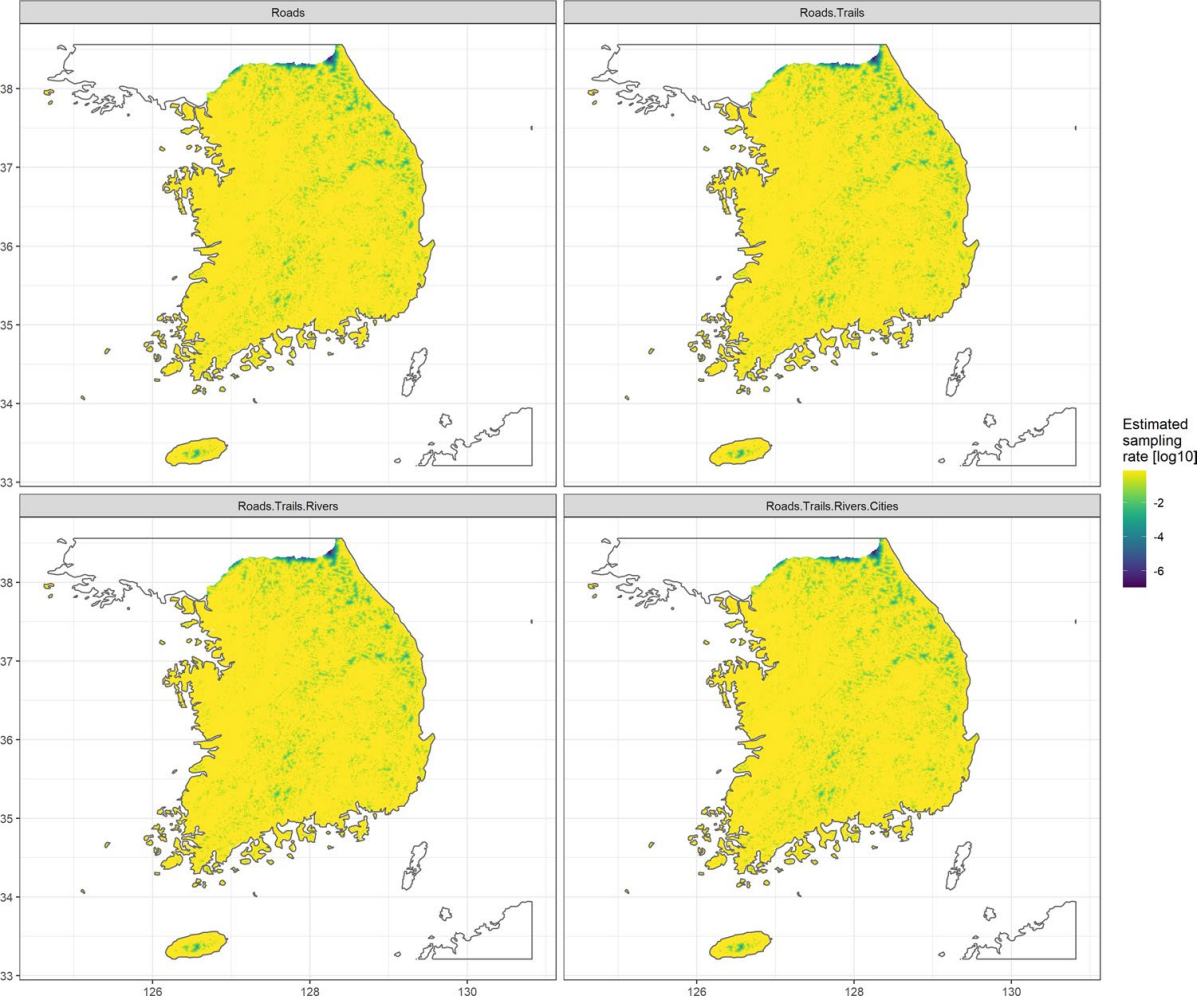
The projection of log-scaled estimated sampling rate by potential biasing factors on the map of South Korea.

To check the potential sampling bias of these raw data, we employed R package sampbias^61^, following the developer’s guidelines. We used roads, mountain trails, rivers, and cities as candidate biasing factors which derived from National spatial data infrastructure portal (http://data.nsdi.go.kr/) and public data portal (https://www.data.go.kr/), respectively. The raster resolution of calculating bias was set to 0.01 degree (~1 km). Because the results from the sampbias analysis did not indicate any significant sampling bias (Figs. 10,11), we did not discuss details in the Results section. The only biasing factor with a high posterior weight was ‘roads’, but the sampling rate depending on the ‘roads’ was consistent, except for a couple of distance points, (Fig. 10) and the projection of estimated sampling rate by biasing factors also showed expected result (relatively low sampling rate for high-elevation sites and the border line with North Korea) throughout South Korea (Fig. 11).

After that, the number of recorded species belonging to each functional group was converted into a ratio of the number of total species within the group for each grid (‘within-grid ratio’, value between 0–1) and displayed on the map. Plotting was conducted using Qgis 3.18.

### Species richness and functional diversity calculation

We calculated species richness as an index of taxonomic diversity^62^. In addition, we calculated functional diversity indices (FRic - functional richness, FEve - functional evenness, FDiv - functional divergence) for amphibians and reptiles, respectively, using the FD package^63^ in R. The distribution of values of each index in each grid were divided into five quantiles that are represented by different colors. Each index was then displayed on the geographic map of South Korea with the same grid size aforementioned.

Due to insufficient survey data, not all grids could have functional diversity values. For the limitations of the binary (i.e., presence/absence) point data, we substituted the ENM value for the number of individuals per species (i.e., species abundance) to make up when calculating functional diversity. We could not perform ENM for *Gekko japonicus*, *Scincella huanrenensis*, and *Sibynophis chinensis*, because of the inadequate number of points, thereby omitting the calculation of biodiversity indices of them. ENM was calculated using Google Earth Engine following Crego *et al*.^64^. We modeled ENM using a machine learning method, random forest with 1,000 decision trees. After spatially thinning point data, we performed 10 block repeated split-sample cross-validation^65,66^. We ran 10 iterations in which the spatial blocks were randomly selected 70% of the data for model fitting and 30% of the data for validation each time. Area Under the Curve of the Receiver Operator Characteristic (AUC) was calculated to verify the accuracy of the model^67^. All species ENM had AUC values of >0.8 (https://hyunkim36.users.earthengine.app/view/enmherptilekor). For predictor variables in ENM, we calculated the percentage cover of trees by MODIS NDVI median value and collected temperature seasonality, annual precipitation, the maximum temperature of the warmest month, and the minimum temperature of the coldest month from the WorldClim BIO database^68^.

The relationship between the biodiversity indices and slope and elevation was confirmed with the average slope and DEM extracted for each grid, and these values were divided into quartiles (SlopeQ. 1^st^: 0.115°–0.973°, SlopeQ. 2^nd^: 1.973°–6.727°, SlopeQ. 3^rd^: 6.727°–12.227°, SlopeQ. 4^th^: 12.227°–19.523°, DEMQ. 1^st^: 0.113–15.855 m, DEMQ. 2^nd^: 15.855–72.325 m, DEMQ. 3^rd^: 72.325–47.675 m, DEMQ. 4^th^: 247.675–987.383 m). Statistical analysis of diversity indices and slope or elevation was performed with Kruskall-Wallis test and Games-Howell post-hoc test in R.

## Data avalability

All data Files are available in figshare^69^. ‘Data file 1_Functional trait amphibians’ and ‘Data file 2_Functional trait reptiles’ are functional trait database. ‘Data file 3_SpeciesWgrid_Fun.Group’ is the species occurrence and functional group data of each grid. ‘Data file 4_Diversity indices distribution’ is shapefile and shows the average functional diversity indices and species richness on each grid.

## Supplementary information

Supplementary Table.

## Data Availability

The code used for analyses in this study can be found at https://github.com/JHKim36/FunctionalDiversisty/blob/main/Herpetofauna_code. ENM results and additional model information can be found at https://hyunkim36.users.earthengine.app/view/enmherptilekor.
