# Supplementary material for: Functional group analyses of herpetofauna in South Korea using a large dataset
Source: Sci Data. 2023 Jan 5;10:15. doi: 10.1038/s41597-022-01924-z (PMC9816106; doi:10.1038/s41597-022-01924-z)
Supplement: Supplementary file 1 — Supplementary Table. [file 41597_2022_1924_MOESM1_ESM.docx]

**Supplementary Table 1.** Species contained in the data analyzed in this study. Data contains taxonomic hierarchy of each species and its record count during the 3^rd^ and 4^th^ National Ecosystem Survey each and in total, and the percentage of area (in pixels) occupied. Taxonomic information followed the 2020 National Species List of the National Institute of Biological Resources for Korea, except *Elaphe schrenckii anomala* which was recorded separately from *Elaphe schrenckii* during the 3^rd^ survey. Although non-native turtles were included in the data, they were excluded for data analyses. Non-native turtles included *Pseudemys concinna* (River cooter), *Pseudemys peninsularis* (Peninsula cooter), and *Mauremys sinensis* (Chinese stripe-necked turtle).

| Class | Order | Family | Genus | Species | Record count (3^rd^) | Record count (4^th^) | Record count (Total) | % of area (in grids) |
| --- | --- | --- | --- | --- | --- | --- | --- | --- |
| Amphibia | Salientia | Bufonidae | *Bufo* | *Bufo gargarizans* | 1,339 | 413 | 1,752 | 62.5 |
| Amphibia | Salientia | Bufonidae | *Bufo* | *Bufo stejnegeri* | 283 | 21 | 304 | 13.0 |
| Amphibia | Salientia | Bombinatoridae | *Bombina* | *Bombina orientalis* | 3,741 | 1,407 | 5,148 | 72.5 |
| Amphibia | Salientia | Microhylidae | *Kaloula* | *Kaloula borealis* | 69 | 109 | 178 | 9.6 |
| Amphibia | Salientia | Ranidae | *Rana* | *Rana uenoi* | 4,434 | 1,618 | 6,052 | 83.8 |
| Amphibia | Salientia | Ranidae | *Rana* | *Rana huanrenensis* | 1,237 | 506 | 1,743 | 38.8 |
| Amphibia | Salientia | Ranidae | *Rana* | *Rana coreana* | 1,797 | 640 | 2,437 | 61.6 |
| Amphibia | Salientia | Ranidae | *Lithobates* | *Lithobates catesbeianus* | 2,630 | 683 | 3,313 | 48.5 |
| Amphibia | Salientia | Ranidae | *Pelophylax* | *Pelophylax nigromaculatus* | 7,039 | 2,659 | 9,698 | 95.3 |
| Amphibia | Salientia | Ranidae | *Pelophylax* | *Pelophylax chosenicus* | 32 | 80 | 112 | 4.2 |
| Amphibia | Salientia | Ranidae | *Glandirana* | *Glandirana rugosa* | 1,920 | 988 | 2,908 | 71.0 |
| Amphibia | Salientia | Hylidae | *Dryophytes* | *Dryophytes japonicus* | 5,696 | 2,254 | 7,950 | 95.7 |
| Amphibia | Salientia | Hylidae | *Dryophytes* | *Dryophytes suweonensis* | 3 | 30 | 33 | 1.1 |
| Amphibia | Caudata | Hynobiidae | *Hynobius* | *Hynobius leechii* | 3,888 | 1,420 | 5,308 | 84.2 |
| Amphibia | Caudata | Hynobiidae | *Hynobius* | *Hynobius yangi* | 3 | 25 | 28 | 0.6 |
| Amphibia | Caudata | Hynobiidae | *Hynobius* | *Hynobius quelpaertensis* | 102 | 63 | 165 | 3.2 |
| Amphibia | Caudata | Hynobiidae | *Onychodactylus* | *Onychodactylus koreanus* | 286 | 72 | 358 | 17.5 |
| Amphibia | Caudata | Plethodontidae | *Karsenia* | *Karsenia koreana* | 13 | 18 | 31 | 2.2 |
| Reptilia | Squamata | Colubridae | *Elaphe* | *Elaphe schrenckii* | 71 | 82 | 153 | 12.1 |
| Reptilia | Squamata | Colubridae | *Elaphe* | *Elaphe schrenckii anomala* | 4 | 0 | 4 | 0.4 |
| Reptilia | Squamata | Colubridae | *Elaphe* | *Elaphe dione* | 1,453 | 736 | 2,189 | 75.9 |
| Reptilia | Squamata | Colubridae | *Oocatochus* | *Oocatochus rufodorsatus* | 641 | 448 | 1,089 | 52.2 |
| Reptilia | Squamata | Colubridae | *Dinodon* | *Dinodon rufozonatum* | 590 | 341 | 931 | 52.0 |
| Reptilia | Squamata | Colubridae | *Amphiesma* | *Amphiesma vibakari* | 175 | 43 | 218 | 15.5 |
| Reptilia | Squamata | Colubridae | *Sibynophis* | *Sibynophis chinensis* | 4 | 7 | 11 | 1.0 |
| Reptilia | Squamata | Colubridae | *Hierophis* | *Hierophis spinalis* | 45 | 13 | 58 | 5.4 |
| Reptilia | Squamata | Colubridae | *Rhabdophis* | *Rhabdophis tigrinus* | 2,827 | 1,355 | 4,182 | 89.7 |
| Reptilia | Squamata | Viperidae | *Gloydius* | *Gloydius ussuriensis* | 1,704 | 744 | 2,448 | 73.6 |
| Reptilia | Squamata | Viperidae | *Gloydius* | *Gloydius brevicaudus* | 757 | 284 | 1,041 | 54.4 |
| Reptilia | Squamata | Viperidae | *Gloydius* | *Gloydius saxatilis* | 107 | 28 | 135 | 11.7 |
| Reptilia | Squamata | Scincidae | *Scincella* | *Scincella vandenburghi* | 422 | 121 | 543 | 27.1 |
| Reptilia | Squamata | Scincidae | *Scincella* | *Scincella huanrenensis* | 15 | 3 | 18 | 1.1 |
| Reptilia | Squamata | Lacertidae | *Takydromus* | *Takydromus amurensis* | 943 | 215 | 1,158 | 41.4 |
| Reptilia | Squamata | Lacertidae | *Takydromus* | *Takydromus wolteri* | 882 | 305 | 1,187 | 43.9 |
| Reptilia | Squamata | Lacertidae | *Eremias* | *Eremias argus* | 21 | 33 | 54 | 3.3 |
| Reptilia | Squamata | Gekkonidae | *Gekko* | *Gekko japonicus* | 0 | 1 | 1 | 0.1 |
| Reptilia | Testudines | Trionychidae | *Pelodiscus* | *Pelodiscus sinensis* | 59 | 5 | 64 | 7.9 |
| Reptilia | Testudines | Trionychidae | *Pelodiscus* | *Pelodiscus maackii* | 4 | 29 | 33 | 7.9 |
| Reptilia | Testudines | Geoemydidae | *Mauremys* | *Mauremys reevesii* | 21 | 18 | 39 | 3.4 |
| Reptilia | Testudines | Emydidae | *Trachemys* | *Trachemys scripta* | 120 | 101 | 221 | 13.7 |
| Reptilia | Testudines | - | - | Non-native turtles | 0 | 8 | 8 | 0.6 |
| Total | 4 | 15 | 25 | 42 | 45,377 | 17,926 | 63,303 |  |
